# 

**Published:** 1893-07

**Authors:** 


### CONTENTS.

ORIGINAL ARTICLES—

A Review of Dr. Senn's Views on Elastic Constriction, by W. H. Elliott, M. D., Savannah, Ga ........ 341

Opthalmia of the New Born, by S. Latimer Phillips, M. D., Savannah, Ga........ 346

Partial Tenotomy—A Radical Cure for Heterophoralgia, by C. H. Peete, M. D., Macon, Ga........ 353

Report of Case, by C. J. Carter, M. D., Lake Park, Ga........ 357

CORRESPONDENCE—

New York Letter ........ 359

SOCIETY REPORTS—

Philadelphia Academy of Surgery. Multiple Fracture of Both Upper Extremities, by William J. Taylor, M. D, Philadelphia, Pa........ 363

A Method of Operating About the Face, by Which Little Blood Enters the Mouth, by W. W. Keen, M. D........ 365

A Case of Appendicitis, by William Hunt, M. D........ 366

BOOK REVIEWS—

Elementary Physiology for Students, by A. T. Schofield, M. D. ; The Uses of Water in Modern Medicine, by Simon Baruch, M. D. ; Cerebral Meningitis, by Martin W. Barr, M. D.; Syllabus of Obstetrical Lectures, by R. C. Norris, A. M., M. D.; Cholera—Its Protean Aspects and Its Management, by A. G. Stockwell, F. Z. S........ 369

The Principles and Practice of Bandaging, by G. G. Davis, M. D........ 370

(Continued on Next Page.)

### CONTENTS—Continued.

Handbook of Massage, by Emil Kleen, M. D.................. 370
Diseases of the Skin, by Chas. C. Ramsom, M. D............. 370
A Treatise on Hygiene and Public Health, by S. F. Murphy.. 370
Notes on the Newer Remedies, their Therapeutic Applications and Modes of Administration, by David Cerna, M. D....... 371
Diseases of the Lungs, Heart and Kidney, by N. S. Davis, Jr., A. M., M. D.................................... 371
Text-Book of Opthalmalogy, by Dr. Ernest Fuchs............ 371

SELECTIONS AND ABSTRACTS—

Gastric and Typhoid Fever.................................. 358
Recovery in a Case of Collapse with Vomiting and Diarrhœa, by the Intravenous Injection of Salt Solution... .......... 362
Treatment of Hydrocele..................................... 362
Internal Urethrotomy, Etc.................................. 372–381

EDITORIALS—

American Medical Association—The Meeting at Milwaukee... 383
Anastomosis Button........ ............................. 385
The National Association of Railway Surgeons............ 386

EDITORIAL NOTES—........................................... 387–388

BOOKS AND PAMPHLETS RECEIVED—............................. 389

PRESCRIPTION DEPARTMENT—................................... 390–391

SPECIAL NOTES—............................................. 392

